# In Vitro and In Vivo Preclinical Testing of Pericyte‐Engineered Grafts for the Correction of Congenital Heart Defects

**DOI:** 10.1161/JAHA.119.014214

**Published:** 2020-02-11

**Authors:** Valeria Vincenza Alvino, Michael Kilcooley, Anita C. Thomas, Michele Carrabba, Marco Fagnano, William Cathery, Elisa Avolio, Dominga Iacobazzi, Mohamed Ghorbel, Massimo Caputo, Paolo Madeddu

**Affiliations:** ^1^ Bristol Heart Institute Translational Health Sciences University of Bristol Bristol Royal Infirmary Bristol United Kingdom

**Keywords:** congenital heart disease, grafts, pericytes, tissue engineering, Animal Models of Human Disease, Autonomic Nervous System, Basic Science Research, Cell Therapy, Vascular Biology

## Abstract

**Background:**

We have previously reported the possibility of using pericytes from leftovers of palliative surgery of congenital heart disease to engineer clinically certified prosthetic grafts.

**Methods and Results:**

Here, we assessed the feasibility of using prosthetic conduits engineered with neonatal swine pericytes to reconstruct the pulmonary artery of 9‐week‐old piglets. Human and swine cardiac pericytes were similar regarding anatomical localization in the heart and antigenic profile following isolation and culture expansion. Like human pericytes, the swine surrogates form clones after single‐cell sorting, secrete angiogenic factors, and extracellular matrix proteins and support endothelial cell migration and network formation in vitro. Swine pericytes seeded or unseeded (control) CorMatrix conduits were cultured under static conditions for 5 days, then they were shaped into conduits and incubated in a flow bioreactor for 1 or 2 weeks. Immunohistological studies showed the viability and integration of pericytes in the outer layer of the conduit. Mechanical tests documented a reduction in stiffness and an increase in strain at maximum load in seeded conduits in comparison with unseeded conduits. Control and pericyte‐engineered conduits were then used to replace the left pulmonary artery of piglets. After 4 months, anatomical and functional integration of the grafts was confirmed using Doppler echography, cardiac magnetic resonance imaging, and histology.

**Conclusions:**

These findings demonstrate the feasibility of using neonatal cardiac pericytes for reconstruction of small‐size branch pulmonary arteries in a large animal model.

**Clinical Perspective**

**What Is New?**

- We have successfully isolated pericytes from the heart of piglets and expanded them to engineer clinical‐grade extracellular matrix scaffolds.
- We have provided evidence of feasibility of using a pericyte cellularized scaffold for reconstruction of the pulmonary artery in a piglet model.

**What Are the Clinical Implications?**

- Currently, repeated interventions are required because of the limited remodeling capacity of the acellular material used for correction of cardiac defects.
- In severe cases, palliation becomes necessary before a definitive surgery correction.
- Cardiac pericytes available from leftover tissue of palliative repair could be used to engineer clinical‐grade matrix conduits ready for implantation at the occasion of definitive correction of the pulmonary artery defect.

## Introduction

Congenital heart disease (CHD) is the most common type of birth defect. Worldwide, 1.35 million babies are born with CHD each year, of which ~5000 are in the United Kingdom alone.^1^, ^2^ One major problem in the correction of congenital cardiac defects is that prosthetic grafts can only remodel through repopulation by invading cells from neighbor tissues and the circulation. This spontaneous reparative process is not rapid enough to prevent a progressive mismatch between the graft and recipient's heart. In severe cases, like tetralogy of Fallot, a palliative “shunt” operation becomes necessary to allow to direct blood flow to the lungs and relieve cyanosis before the definitive correction. This temporal window may provide a scope for expanding cardiac cells from surgery leftovers to generate living prostheses. Prostheses endowed with immediate growing capacity ahead of implantation could be better suited to match the rapid growth of a baby's heart than the currently available acellular grafts.

Our previous study has shown that leftovers of palliative surgery can be used to isolate and expand a population of cardiac stromal cells with the characteristics of pericytes.^3^ We have proposed that cardiac pericytes (CPs) could be used to engineer prosthetic grafts for the definitive correction of right ventricle outflow defects.^3^ Compared with competitive solutions, such as vein‐derived autologous endothelial cells (ECs) or peripheral blood‐derived endothelial progenitor cells,^4^, ^5^, ^6^ CPs have the advantage of being tissue specific and capable of differentiating into vascular smooth muscle cells and producing extracellular matrix (ECM) proteins.^3^ Moreover, CPs may favor graft endothelialization through their powerful angiocrine secretome.^3^ In an initial manufacturing attempt, we seeded human neonatal CPs onto CorMatrix, a decellularized porcine ECM clinically approved for use in cardiac surgery.^3^ After 3‐week culture in a flow bioreactor, the conduit was colonized by the proliferating seeded CPs.

To make a step forward in the translational pathway, we have now designed a new study aimed at testing the feasibility of implanting CP‐engineered conduits in piglets using the same procedure utilized to reconstruct branch pulmonary arteries (PAs) in CHD patients. Human CPs represent the final cellular product. However, preclinical testing of human cells in piglets would require early chronic immunosuppression, which could result in confounding and ethically unacceptable adverse effects.^7^, ^8^, ^9^ As a surrogate, we used allogeneic CPs isolated from littermates of the recipient piglet after demonstration of similarities with neonatal human CPs.

## Methods

The article adheres to the American Heart Association journals’ implementation of the Transparency and Openness Promotion Guidelines. In accordance with this policy, authors will make the data, methods used in the analysis, and materials used to conduct the research available to any researcher for purposes of reproducing the results or replicating the procedure.

### Ethics

Institutional review board approval for the study was obtained, according to the guidelines noted in the Journal Instructions to Authors. Animal experiments were performed in accord with institutional guidelines and followed the principles stated in the *Guide for Care and Use of Laboratory Animals* published by the National Institutes of Health in 1996 and in the Animals (Scientific Procedures) Act published in 1986. The protocol was covered by the UK Home Office ethical approval PPL 30/3019 and PF6E6335D. The report of experimental data follows the Animal Research: Reporting of In Vivo Experiments (ARRIVE) guidelines.^10^

### Collection of Cardiac Tissue for CP Isolation

Cardiac tissue and peripheral blood were collected from 4‐week‐old large white/Landrace piglets for isolation/expansion and characterization of swine CPs (sCPs) and peripheral blood mononuclear cells, respectively. Sample attribution to experimental analyses is provided in Table S1.

### In Situ Immunohistochemistry and Immunocytochemistry Characterization of sCPs

Details of the methodology used for immunofluorescent microscopy studies are reported in Data S1. The list and source of antibodies are reported in Table S2.

### Production of sCP Stocks

Isolation and expansion of sCPs were performed using an adaptation of the good manufacturing practice–compliant standard operating procedure previously used on human neonatal hearts (details in Data S1).^3^ At passage 2, cells were split for further expansion or generation of frozen stocks.

### Assessment of sCP Characteristics

Cell were studied at passage 5/6, using from 3 to 7 biological replicates (run in triplicate), unless otherwise specified. *Antigenic profile:* Immunofluorescence microscopy (N=7) and flow cytometry analyses (N=3) were performed using the procedures and antibodies described in Data S1 and Tables S2 and S3. *Viability, growth curves, and doubling time:* Three fresh or frozen‐thawed sCP lines were seeded onto a 6‐well plate at a density of 3000/cm.^2^ They were detached at days 4, 5, 6, 7, and 8 of culture and counted using trypan blue (Thermo Fisher Scientific, Loughborough, UK). *Clonogenic assay:* The test was performed on 2 sCP lines at passage 3, using a motorized device connected to the flow cytometric sorter (Cyclone; Beckman Coulter, Brea, CA). A comparative assay between fresh and frozen‐thawed sCPs was performed to assess whether both conditions allow for the generation of clones. *Expression of angiogenic factors:* Quantitative PCR was performed on cells (N=5 biological replicates) cultured under normoxia (21% oxygen) using a Quant Studio 6 Flex Real‐Time PCR system (Applied Biosystems, Foster City, CA). mRNA expression level was determined using the 2‐ΔCt method. Swine pulmonary artery ECs (sPAECs) were used as control. Taqman probes used in these studies are reported in Table S4. *Secretion of angiogenic factors:* Dedicated antihuman ELISA kits (R&D Systems, Minneapolis, MN, US) were used to measure immunoreactive levels of VEGF‐A (vascular endothelial growth factor A), ANG1 (angiopoietin 1), ANG2 (angiopoietin 2), and FGF‐2 (fibroblast growth factor 2) proteins in conditioned media (CM) from sCPs, which were cultured under normoxia (N=4 biological replicates). *Endothelial network formation:* The capacity of cells to form networks on Matrigel was assessed using sCPs or sPAECs alone or both in coculture (N=4 biological replicates). In addition, network formation capacity of sPAECs was assessed following stimulation with sCP‐CM or unconditioned media. *Chemotactic activity*: We tested the capacity of the sCP‐CM (N=4 biological replicates) to induce the migration of sPAECs in a transwell cell‐culture system. Endothelial cell basal medium‐2 basal medium or endothelial cell basal medium‐2 supplemented with 100 ng/mL of swine recombinant VEGF‐A were used as controls. In separate assays, an antagonist of Tie2 kinase receptor was used to contrast the effect of sCP‐CM on migration. *Endothelial proliferation*: The capacity of sCPs and sCP‐CM (N=4 biological replicates) to promote sPAEC proliferation was assessed by the Click‐iT EdU Cell Proliferation Kit for Imaging, Alexa Fluor 488 dye (Thermo Fisher Scientific). sPAECs maintained in unconditioned medium were used as a control.

### Static Culture of sCPs on CorMatrix

Pieces of CorMatrix ECM were primed with endothelial cell growth medium‐2 media for 48 hours and then seeded with sCPs (passage 5; 20 000/cm^2^) and cultured for 5 days in a 48‐well plate. The CM was then collected and the CorMatrix samples were cut in 3 pieces: One was fixed and paraffin‐embedded for histological studies; the other 2 were frozen for optimal cutting temperature embedding and for RNA and protein analyses. Eight biological replicates were examined, unless differently specified.

### Dynamic Culture of sCPs on CorMatrix

After completion of the 5‐day static culture, sCP‐seeded CorMatrix was matured in a 3‐dimensional CulturePro Bioreactor (TA Instruments, New Castle, DE, US). Nonseeded CorMatrix was used as control. Seven and 14 days later, the CM was collected, and the conduits were unstitched and cut in 3 pieces as for static culture. Further samples were also taken for mechanical and cell viability studies. The list of reagents and specific use of derived sCP lines in the CorMatrix studies are reported in Table S5.

### Characterization of Cellularized CorMatrix Conduits

*Histological assessment of the conduit structure:* To obtain a general layout of the cell and interstitial collagen distribution within the CorMatrix conduit, frozen sections were stained with hematoxylin and eosin, elastic tissue van Gieson, and Mallory's trichrome. *Viability and apoptosis:* After detachment from the CorMatrix conduit by enzymatic digestion, cell viability was determined with trypan blue and apoptosis with ApopTag Red. In addition, cell viability was assessed in situ using a Viability/Cytotoxicity immunofluorescent kit (Thermo Fisher Scientific). Saponin‐treated samples were used as positive controls for cell death. *Proliferation:* After permeabilization, samples were stained with antiswine Ki67 primary antibody (Abcam, Cambridge, UK). *Expression of mural cell markers:* Sections were incubated with an antiswine neural/glial antigen 2 (NG2) primary antibody or with antihuman and ‐swine α‐SMA (α‐smooth muscle actin; Sigma‐Aldrich, Gillingham, UK), calponin (Abcam), transgelin (Santa Cruz Biotechnology, Peaslake, UK), smoothelin (Santa Cruz Biotechnology), and smooth muscle myosin heavy chain (Abcam). Then, secondary antibodies were added on the sections for 1 hour. *Analysis of collagen secretion:* Levels of soluble collagen 1 in the CM of cellularized conduits were assessed using an antihuman ELISA kit (R&D Systems, Minneapolis, MN, US). *Mechanical tests of CorMatrix conduits:* Elastic modulus, maximum tensile stress, and strain at rupture of unseeded and cellularized conduits (N=4 biological replicates) were measured using an Instron 3343 device. A swine left pulmonary artery (LPA) specimen served as control.

### In Vivo Feasibility Study in Piglet Model of LPA Reconstruction

Two 9‐week‐old littermate Landrace female piglets, under general anesthesia and neuromuscular blockade, underwent LPA resection to accommodate the conduit (~10 mm long and ~6 mm in diameter), as previously reported^11^ and described in detail in Data S1. One piglet received a conduit cellularized with sCPs from a sister piglet and cultured under static (5 days) and dynamic conditions (7 days). The other piglet was implanted with an unseeded conduit. Animals recovered under intense postoperative monitoring for the initial 24 hours. Imaging studies were performed using a 2‐dimensional Doppler echocardiography system (VividQ; GE Healthcare, Cardiff, UK) and a cardiac magnetic resonance 3‐Tesla scanner (Siemens Healthcare, Erlangen, Germany) at baseline and 4 months after implantation, with further echocardiography at 1, 2, and 3 months postsurgery. Then, swine were euthanized, and the grafted LPA was harvested. Tissue was snap frozen, fresh frozen in optimal cutting temperature compound, or fixed in 4% paraformaldehyde before paraffin inclusion. Sections were used for histology, immunohistochemistry, and morphometric analyses.

### Statistical Analyses

The Student *t* test was used when comparing 2 groups and ANOVA with post hoc when comparing >2 groups. Values are expressed as means±SEM or SD. Probability values (*P*) <0.05 were considered significant. Results from the in vivo feasibility study are reported in a descriptive format.

## Results

### Immunohistochemical Localization of sCPs

**Figure 1:**
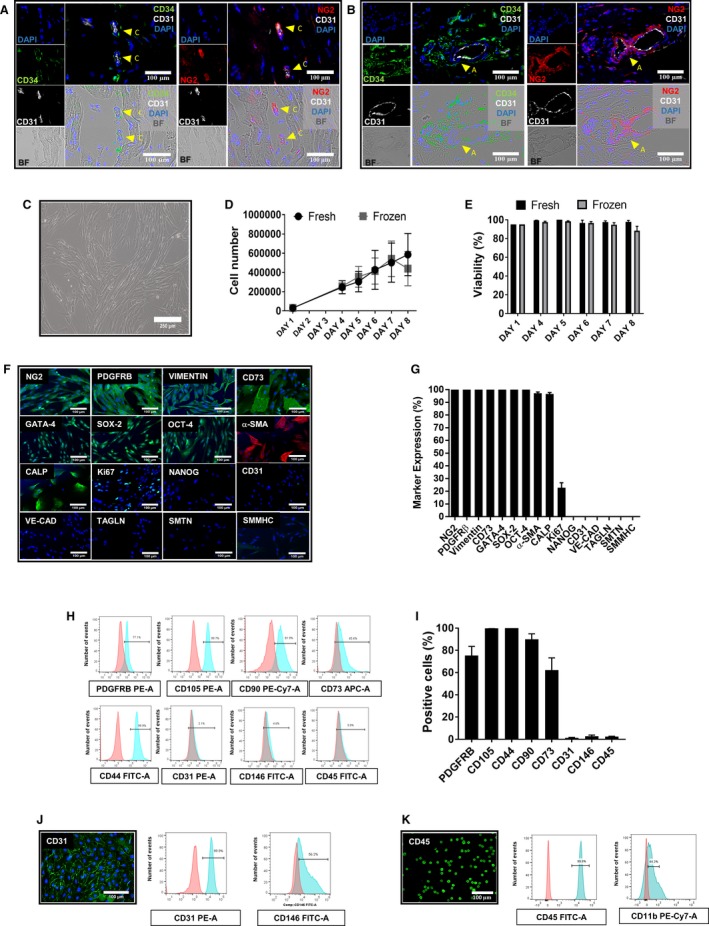
Characterization of swine cardiac pericytes. (**A** and **B**) Perivascular localization of sCPs in situ. Immunofluorescence images showing the localization of CD31^–^/CD34^+^/NG2^+^ CPs in swine hearts around capillaries (**A**) and arterioles (**B**). Inserts showing CD34 labeled in green fluorescence, NG2 in red, and CD31 in white; nuclei are recognized by blue fluorescence of DAPI. Arrows indicate CPs around capillaries and an arteriole. Images taken at 200× magnification. **C**, Phase contrast microscopy image of sCPs displaying spindle‐shape features (magnification, ×100). (**D** and **E**) Graphs showing the growth curve (**D**) and viability (**E**) of 3 sCP lines that were seeded at 3000 cells/cm^2^ at day 1 and detached and counted at days 4, 5, 6, 7, and 8 of culture. **F**, Immunofluorescence microphotographs showing the expression of neural/glial antigen 2 (NG2) and platelet‐derived growth factor receptor beta (PDGFRB), vimentin, CD73, cardiac transcriptional factor GATA‐binding protein 4 (GATA‐4), and the stemness markers, sex determining region Y‐box 2 (SOX‐2) and octamer‐binding transcription factor 4 (OCT‐4). Cells are negative for NANOG and the endothelial cell markers, vascular endothelial‐cadherin (VE‐cadherin) and CD31. sCPs express alpha‐smooth muscle actin (α‐SMA) and calponin (CALP) and are negative for transgelin (TAGLN), smoothelin (SMTN), and smooth muscle myosin heavy chain (SMMHC). Expression of Ki67 is indicative of proliferating cells. DAPI (blue) identifies nuclei. Scale bars=100 μm. **G**, Values in bar graph represent the mean±SEM of 7 biological replicates from immunocytochemistry studies. Fluorescence was normalized by nuclei count. (H and I) Flow cytometry analysis of 3 sCP lines at P5. **H**, Representative graphs for each surface marker; negative control staining profiles are shown by the red histograms, whereas specific antibody staining profiles are shown by light blue histograms. **I**, Bar graph shows the mean±SEM values of 3 sCP lines. (**J** and **K**) Immunofluorescent images and flow cytometry histograms are also shown for swine pulmonary artery endothelial cells (PAECs) (**J**) and peripheral blood mononuclear cells (PBMNCs; **K**). APC indicates allophycocyanin; BF, bright field; CD, cluster of differentiation; DAPI, 4′,6‐diamidin‐2‐fenilindolo; FITC‐A, fluorescein‐area; PE‐A, phycoerythrin‐area; PE‐Cy7, phycoerythrin–cyanine 7; sCP, swine cardiac pericytes.

Multiple target immunohistochemistry was used for cell‐type localization in cardiac tissue (Figure 1). CD31^pos^/CD34^pos^ ECs were typically found around the lumen of capillaries and arterioles. Moreover, we identified CD31^neg^/CD34^pos^/NG2^pos^ cells around capillaries (Figure 1A) and especially within the external lamellae of arterioles (Figure 1B).

### Antigenic Profile of Expanded sCPs

To isolate and generate stocks of sCPs from piglets hearts, we used a modified version of the standard operating procedure previously used on leftovers of CHD reconstructive surgery,^3^ the main changes being the substitution of fetal bovine serum with swine heat‐inactivated serum (as a constituent of the growth medium) and swine gelatin (as a coating material of culture dishes). For the purpose of the present study, we have successfully isolated and expanded 15 CP lines from pieces of piglet cardiac ventricles as small as 0.01 g in weight (lowest limit of success).

We demonstrated that expanded cells have a typical spindle‐shaped morphology (Figure 1C). Cells grew quickly in culture with an average doubling time of 44.5  (using fresh cells) and 41.7 hours (using thawed cells). From the initial seeding number (30 000 cells), sCPs reached the final counts of ~600 000 (fresh) and ~500 000 cells (thawed) at 8 days of culture (Figure 1D). Viability of expanded cells was consistently >90% in all the samples examined (Figure 1E).

Immunocytochemistry analyses illustrated in Figure 1F and 1G confirmed that expanded cells express the mesenchymal markers, NG2, PDGFRβ (platelet‐derived growth factor receptor beta), vimentin, and ecto‐5‐nucleotidase (CD73). Moreover, they were positive for the cardiac transcriptional binding factor, GATA‐binding protein 4, and the transcription factors, (sex determining region Y)‐box 2 and octamer‐binding transcription factor 4, but negative for nanog homeobox (NANOG) and the endothelial markers, vascular endothelial‐cadherin and CD31. When looking at markers that identify vascular smooth muscle cells, immunocytochemistry revealed that sCPs express α‐SMA and calponin. Moreover, positive expression for Ki67 confirmed their proliferative activity. Using flow cytometry, we demonstrated that, like human CPs, sCPs abundantly express the pericyte marker, PDGFRβ, mesenchymal markers CD105, CD90, CD44, and CD73, while being negative for CD146, CD31, and the hematopoietic marker, CD45 (Figure 1H and 1I). sPAECs (Figure 1J) and peripheral blood mononuclear cells (Figure 1K) were used as positive controls for expression of CD31 and CD146 (endothelial markers) and CD45 and CD11b (hematopoietic markers) in flow cytometry and immunocytochemistry studies.

### Clonogenic Potential of Expanded sCPs

**Figure 2:**
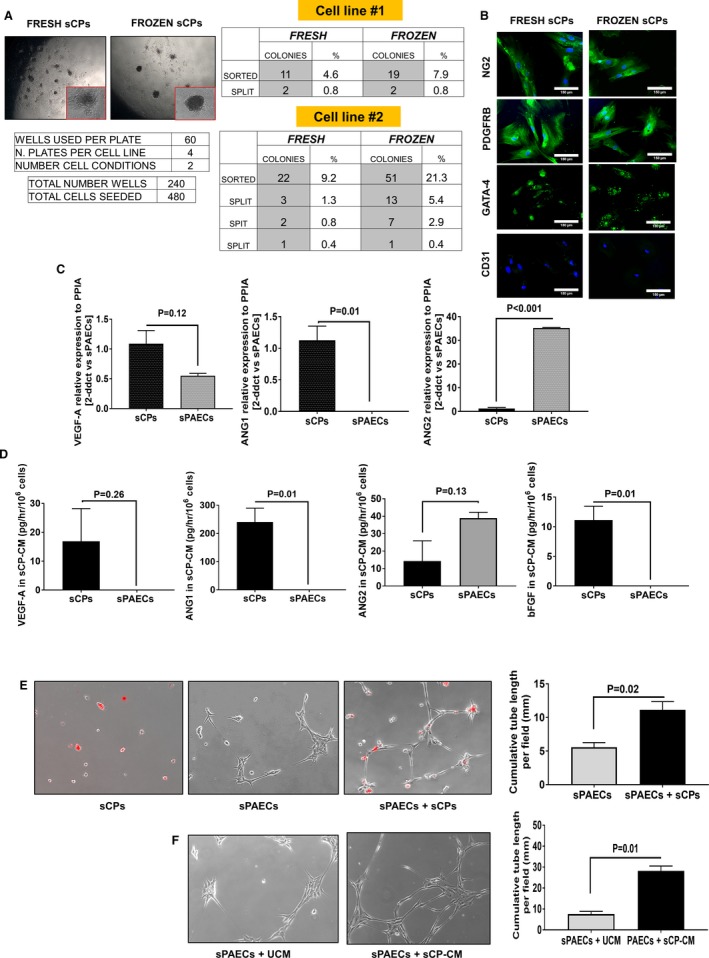
Functional features of swine cardiac pericytes. (**A** and **B**) Clonogenic assay of 2 sCPs lines. **A**, Images showing the colony formation after 2 weeks from sorting. Single CPs were deposited to the bottom of a 96 multiwell plate. As indicated in the tables, 2 fresh cell lines formed an average of 6.9% small colonies and 2 thawed cell lines gave rise to an average of 14.6% colonies. The second cell line formed new colonies after splitting. **B**, Immunofluorescent images of colonies from fresh and thawed cells confirm the positivity for NG2, PDGFRB, and GATA‐4 and the negativity for CD31 antigen. **C**, Bar graphs show expression of VEGF‐A, ANG1, and ANG2 in sCP and sPAEC lysates. Values are means±SEM, N=5 per each group. **D**, Bar graphs show VEGF‐A, ANG1, ANG2, and bFGF protein levels in the CM of sCP and sPAECs. Values are means±SEM, N=4 per each group. (**E** and **F**) Direct and paracrine angiogenic activity of sCPs in a Matrigel assay. **E**, Representative phase‐contrast images (100× magnification) of networks formed by sPAECs and sCPs cultured alone or in combination (sCPs to sPAECs at a 2:5 ratio) on Matrigel substrate for 6 hours. sCPs were stained with the long‐term cell tracker, chloromethylbenzamido (Dil; red fluorescence), to assess the ability to cooperate with sPAECs in forming network structures. Bar graph showing the cumulative tube length per field. **F**, Representative phase‐contrast images of network formation by sPAECs cultured on Matrigel in the presence of unconditioned media (UCM) or sCP conditioned media (CM) for 6 hours. Bar graph showing the cumulative tube length per field. Values are means±SEM, N=4 per each group. ANG1 indicates angiopoietin 1; ANG2, angiopoietin 2; bFGF, basic fibroblast growth factor; GATA‐4, cardiac transcription factor‐4; NG2, neural/glial antigen 2; PDGFRβ, platelet‐derived growth factor receptor beta; PPIA, peptidylprolyl isomerase A; sCPs, swine cardiac pericytes; sCP‐CM, swine cardiac pericyte‐conditioned media; sPAECs, swine pulmonary artery endothelial cells; VEGF‐A, vascular endothelial growth factor A.

Two biological replicates were sorted as single cells and cultured in 96‐well plates (Figure 2A). Two weeks later, 4.6% and 9.2% of freshly processed cells formed colonies. The same cell lines underwent the clonogenic assay after a cycle of freezing and thawing. In this experiment, colonies were generated by the 2 cell lines with a frequency of 7.9% and 21.3%, respectively. Of these primary colonies, 2 (0.8%) and 3 (1.3%) from fresh cell lines and 2 (0.8%) and 13 (5.4%) from thawed cell lines could be further expanded in culture. Figure 2B shows that clonogenic cells maintained the original phenotype as assessed by immunocytochemistry.

### Angiocrine Properties of Expanded sCPs

qPCR analysis of angiogenic gene trascripts in 5 sCP lines showed that these cells express more *VEGF‐A* and *ANG1* , but less *ANG2*, than control sPAECs (Figure 2C). A similar pattern was observed comparing levels of those angiogenic factors in the CM (Figure 2D). In addition, FGF‐2 protein levels were remarkably higher in CM of sCPs compared with sPAECs (Figure 2D). This angiocrine profile resembles the described paracrine phenotype of human CPs.^3^ Both sCPs (Figure 2E) and their CM (Figure 2F) could enhance the network‐forming capacity of sPAECs.

### Chemotactic Activity of Expanded sCPs on sPAECs

**Figure 3:**
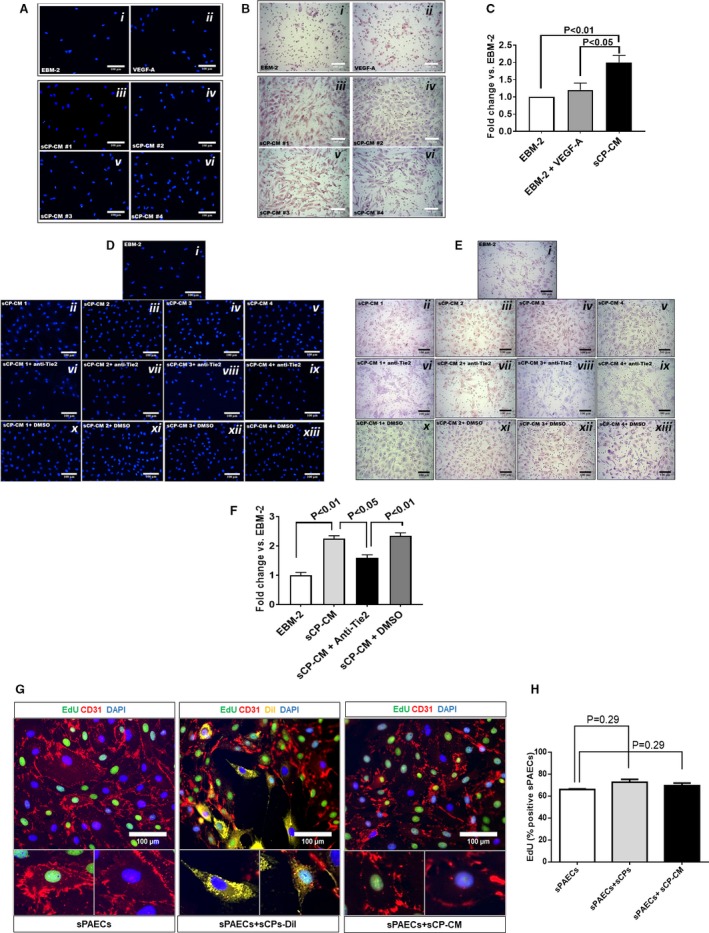
Chemotactic activity of factors secreted by swine cardiac pericytes. (**A** through **C**) In a transwell migration assay, sCP‐CM enhanced the migration of sPAECs. Representative images of migrated cells stained by DAPI (**A**) or Giemsa (**B**) following stimulation with EBM‐2 (*i*), VEGF‐A (*ii*, 100 ng/mL), or CM from 4 sCP lines (*iii*–*vi*). Images acquired using a 200× magnification. (**C**) Bar graph showing the fold change of migrated cells vs EBM‐2 basal media. N=4, data are mean±SEM. (**D** through **F**) Effect of Tie‐2 inhibitor (7.5 μmol/L) on the chemotactic activity of sCP‐CM. Representative images of migrated cells stained by DAPI (**D**), or Giemsa (**E**) following stimulation with EBM‐2 (*i*), CM from 4 sCP lines (*ii*–*v*), or CM from the same sCP lines added with a Tie‐2 antagonist (*vi*–*ix*) or its vehicle (*x*–*xiii*, DMSO). Images acquired using a 200× magnification. **F**, Bar graph showing the fold change of migrated cells vs EBM‐2 basal media. Data are mean±SEM. Representative immunofluorescent images of proliferating sPAECs following coculture with sCPs and sCP‐CM. (**G**) sPAECs are stained with anti‐CD31 (red fluorescence) and sCPs with the long‐term cell tracker, Dil (yellow fluorescence). Images acquired using 200× magnification. (**H**) Bar graph displaying the percentage of EdU^+^ sPAECS following stimulation with sCPs and sCP‐CM. N=4, data are mean±SEM. CD indicates cluster of differentiation; Dil, chloromethylbenzamido; DMSO, dimethyl sulfoxide; EBM‐2, endothelial cell basal medium‐2; EdU, 5‐ethynyl‐2′‐deoxyuridine; sCP‐CM, swine cardiac pericyte–conditioned media; sPAECs, swine pulmonary artery endothelial cells; Tie2, tyrosine kinase 2; VEGF‐A, vascular endothelial growth factor A.

Next, we examined whether factors secreted by sCPs may exert chemoattractant effects on sPAECs, a property instrumental to the promotion of in vivo endothelialization of the graft. Results of a transwell migration assay, where migrated cells were examined using 4′,6‐diamidin‐2‐fenilindolo or Giemsa staining, revealed that sCP‐CM increased the rate of sPAEC migration by ≈2‐fold compared with endothelial cell basal medium‐2 basal medium or endothelial cell basal medium‐2 supplemented with VEGF‐A (Figure 3A through 3C). Interestingly, the addition of an anti‐Tie2 kinase receptor antagonist inhibited the chemotactic effect of sCP‐CM, thus suggesting that ANG1 signaling is involved in sPAEC attraction (Figure 3D through 3F).

### Proliferation of sPAECs

In addition, we tested whether CPs and their secretome may stimulate sPAEC proliferation (Figure 3G through 3H). Results indicate that the abundance of 5‐ethynyl‐2′‐deoxyuridine–positive sPAECs was not increased by coculturing them with sCPs or exposing them to the sCP‐CM (72.8±2.6% and 69.6±2.3% versus 66.2±2.3% in sPAECs alone; *P*=0.29 for both comparisons). Considering these data in light of the Matrigel and transwell assays results, we speculate that sCPs exert proangiogenic effects through stimulation of sPAECs migration and stabilization of newly formed sPAEC networks, which would be otherwise subjected to spontaneous regression in the Matrigel assay.

### Incorporation of sCPs in a Clinical‐Grade Conduit

Next, we seeded sCPs (20 000 cells/cm^2^) on CorMatrix, a clinically approved graft material, and cultured the seeded and control unseeded conduits for 5 days under static conditions. In additional experiments, the 2 systems were transferred from the static condition to a flow bioreactor for a period of 1 or 2 weeks of dynamic culture.

**Figure 4:**
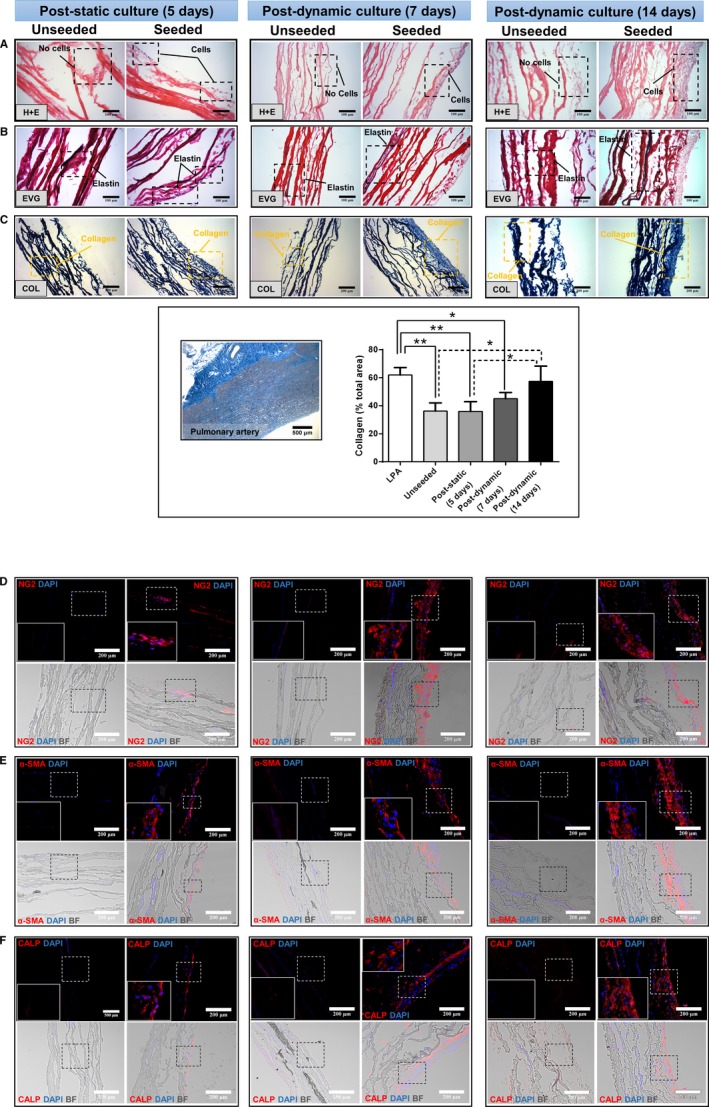
Histology and immunohistochemistry of CorMatrix grafts seeded with swine cardiac pericytes. (**A**) H&E staining. (**B**) Elastin staining with van Gieson, (**C**) Collagen staining with Azan Mallory and quantification of grafts and LPA. Immunofluorescence microscopy images of (**D**) NG2, (**E**) α‐SMA, and (**F**) calponin. Microphotographs were collected from samples collected after 5 days of static culture and at 7‐ and 14‐days culture in the bioreactor. Unseeded control samples are also shown for comparison. BF indicates bright field; CALP, calponin; COL, collagen; DAPI, 4′,6‐diamidin‐2‐fenilindolo; EVG, elastic fibers Van Gieson; H&E, hematoxylin and eosin; LPA, left pulmonary artery; NG2, neural/glial antigen 2; α‐SMA, α‐smooth muscle actin.

Figure 4 shows representative images from the histology and immunohistochemistry analyses performed on conduits harvested at the end of each of the above culture stages. Staining with hematoxylin and eosin documented that seeded cells adhered to the surface of the conduit, forming a thicker layer at 14 days postdynamic culture as compared with poststatic culture (Figure 4A). In seeded conduits, fibers of elastin (van Gieson; Figure 4B) and collagen (azan Mallory; Figure 4C) were clearly detected in proximity of the cell‐seeded area and across the matrix, with the collagen signal intensity being stronger as compared with unseeded conduits. A freshly collected swine LPA was used for comparison. The bar graph shows that there was a progressive increase in collagen content throughout the culture period of the cellularized graft, which peaked at 14 days of the dynamic conditioning in the bioreactor (Figure 4C). At this stage, the initial gap in collagen content between the native CorMatrix and LPA was almost completely abrogated. As expected, soluble collagen was undetectable in the CM of control unseeded CorMatrix. In contrast, secreted soluble collagen averaged 22.4 ± 4.2 ng/mg of protein in the CM collected from cellularized CorMatrix (data not shown). These findings confirm that CPs actively produce ECM proteins (collagen) and maintain this property once incorporated into the conduit.

Next, we verified whether sCPs maintain their original phenotype or acquire additional markers after culture of the conduit under static conditions and subsequent application of flow in the bioreactor. As expected, control unseeded conduits did not show any positive staining for the studied markers, thus confirming these to be acellular. Seeded conduits collected at the end of the static culture showed few NG2‐expressing cells, with this antigenic cell phenotype being remarkedly more abundant after application of flow, but maintaining the original location at the seeded surface of the conduit (Figure 4D). A similar pattern was observed for the other natively expressed antigens, α‐SMA (Figure 4E) and calponin (Figure 4F), whereas cells in the conduits were negative for transgelin, smoothelin, and smooth muscle myosin heavy chain, thus indicating that they did not acquire a mature muscular phenotype with transfer from plastic into the conduit (data not shown).

**Figure 5:**
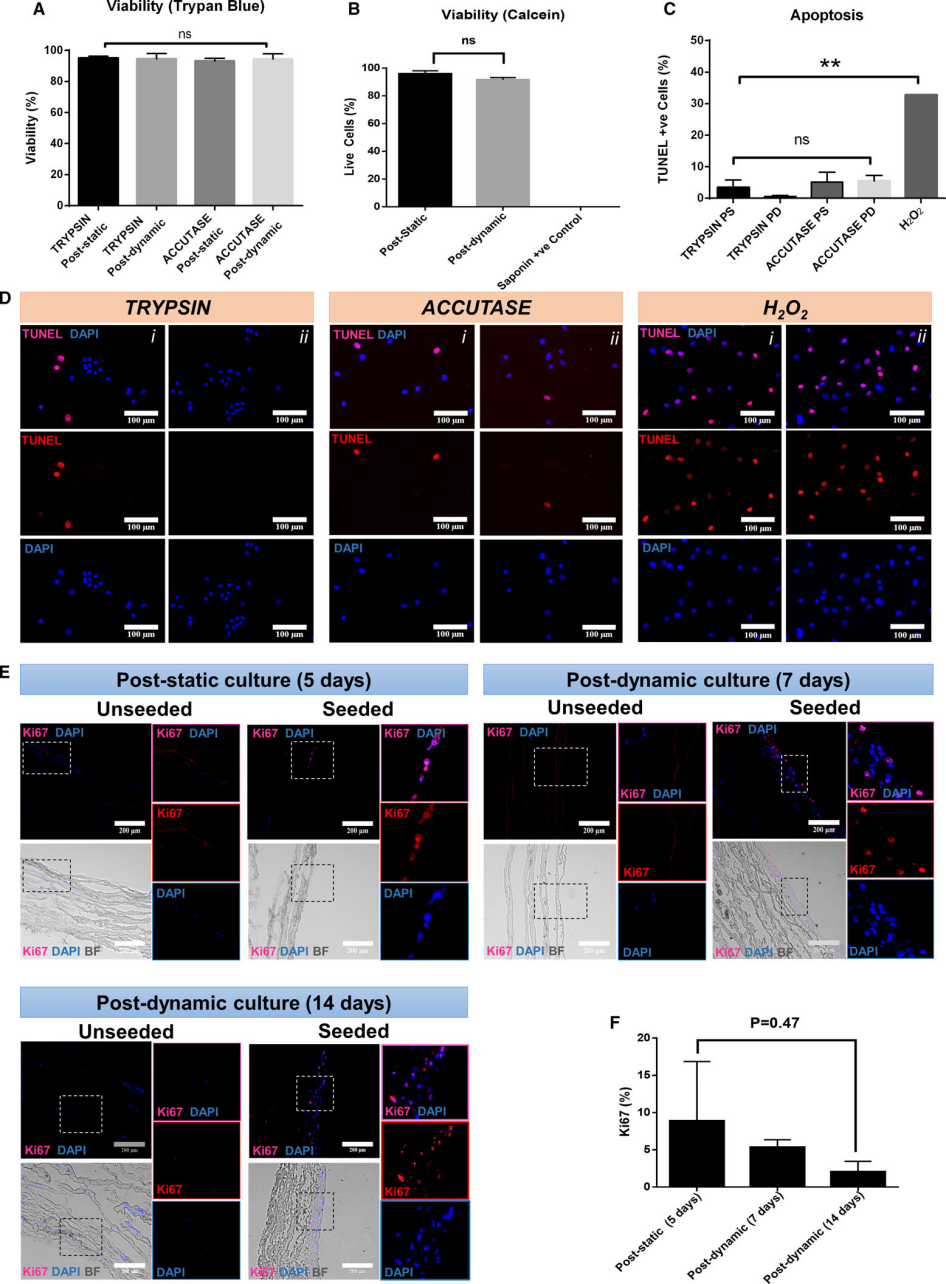
Cell viability and proliferation of CorMatrix grafts engineered with swine cardiac pericytes. **A**, High viability of sCPs detached from the matrix using 2 enzymatic methods at the end of static and dynamic culture. **B**, The data were confirmed using Calcein staining of the grafts. Saponin was used as a control cytotoxic agent. **C** and **D**, Apoptosis was assessed by the TUNEL assay. Bar graph showing results (**C**) and representative microscopic images displayed as single and merged channels of 2 different fields in the same sample (*i*–*ii*, blue=DAPI; red=TUNEL; merge=DAPI/TUNEL) (**D**). Data are mean±SEM of 4 biological replicates. **E**, Representative images of proliferation assessed by Ki67 staining. Fluorescent images are displayed as single and merged channels. No staining was detected in unseeded grafts in the different experimental conditions. **F**, Graphs showing proliferation following static and dynamic conditions. BF indicates bright field; DAPI, 4′,6‐diamidin‐2‐fenilindolo; PD, postdynamic; PS, poststatic; TUNEL, terminal deoxynucleotidyl transferase dUTP nick end labeling; +ve control, positive control.

Viability was assessed in situ with a viability/cytotoxicity immunofluorescence kit as well as using trypan blue after cell detachment from the matrix, using trypsin or accutase, and cell cytospin. Positive controls were treated with saponin or H_2_O_2_. Cells showed a consistently high viability (>90%) in situ and after detachment with both enzymatic methods and length of conditioning time (Figure 5A and 5B). Results of terminal deoxynucleotidyl transferase dUTP nick end labeling assays documented low apoptosis levels (<7%; Figure 5C and 5D). Quantitative analysis of cell proliferation revealed that 8.9±8.0% of total cells expressed Ki67 at 5 days of static culture. Frequency of proliferating cells decreased following dynamic culture (5.4±1.0% and 2.1±1.4% Ki67‐positive cells at 7 and 14 days, respectively; Figure 5E and 5F). However, because of large variability of the measure, the change in proliferation did not reach statistical significance.

### Mechanical Properties of Conduits Engineered With sCPs

**Figure 6:**
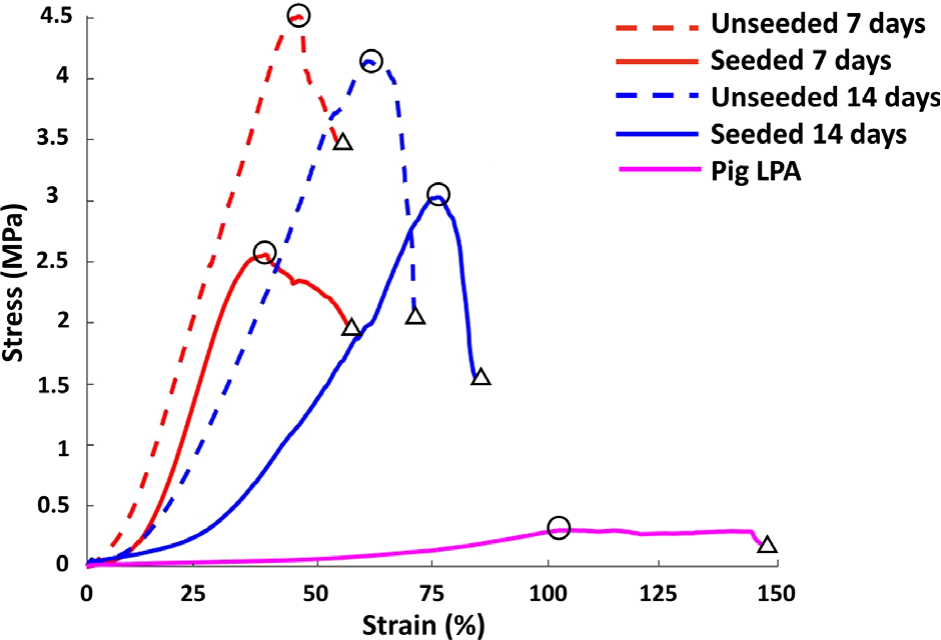
Stress‐strain curves. A representative line graph showing LPA and CorMatrix mechanical properties in the different experimental conditions. Circles within the lines indicate maximum stress values, and triangles indicate the strain at rupture. Average values of the different biological replicates are reported in Table 1. LPA indicates left pulmonary artery.

Seeded and unseeded conduits (4 biological replicates, each one with 3 internal replicates) were tested after 7 and 14 days of maturation in the bioreactor. Representative curves and cumulative data of the relation between stress and strain are reported in Figure 6 and Table 1. Analysis of tensile tests at day 7 displayed a 1.8‐fold reduction in maximum stress for seeded conduits compared with the unseeded ones (*P*<0.05), whereas strain at breaking point and Young's modulus did not differ between groups. The conditioning period of 14 days showed a significant decrease in stiffness, demonstrated by the reduction of Young's modulus (1.7‐fold versus unseeded; *P*<0.05) and an increase in strain at maximum load (1.2‐fold versus unseeded; *P*<0.05), whereas there was no difference in maximum stress. Thus, cellularization reduced the mechanical mismatch between native CorMatrix and LPA, supporting the feasibility of implantation with a lower risk of failure.

**Table 1:** Mechanical Properties of CorMatrix Grafts and Swine LPA

Conditioning Period	Samples	Young's Modulus (MPa)	Maximum Stress (MPa)	Strain at Rupture (%)
7 days	Unseeded	17.7±2.1	4.2±0.5	55±6
	Seeded	13.2±2.6	2.4±0.3*	56±4
14 days	Unseeded	15.5±2.1	4.2±0.6	69±1
	Seeded	8.9±1.5*	3.1±0.7	83±4* ^**,**^ †
	LPA	0.55±0.07	0.28±0.09	151±12

Values are means±SE, N=4 biological replicates, each one with 3 internal replicates. LPA indicates left pulmonary artery.

*P<0.05 vs unseeded grafts within each time point; ^†^ *P*<0.01 vs corresponding group (seeded or unseeded) harvested at 7 days of conditioning in the bioreactor.

### In Vivo Feasibility Study

Finally, we performed a feasibility study where two 4‐week‐old sister weaner piglets underwent LPA surgical replacement with seeded or unseeded CorMatrix conduits, respectively. Both animals survived the experimental period and showed similar growing curves.

- LPA imaging

**Figure 7:**
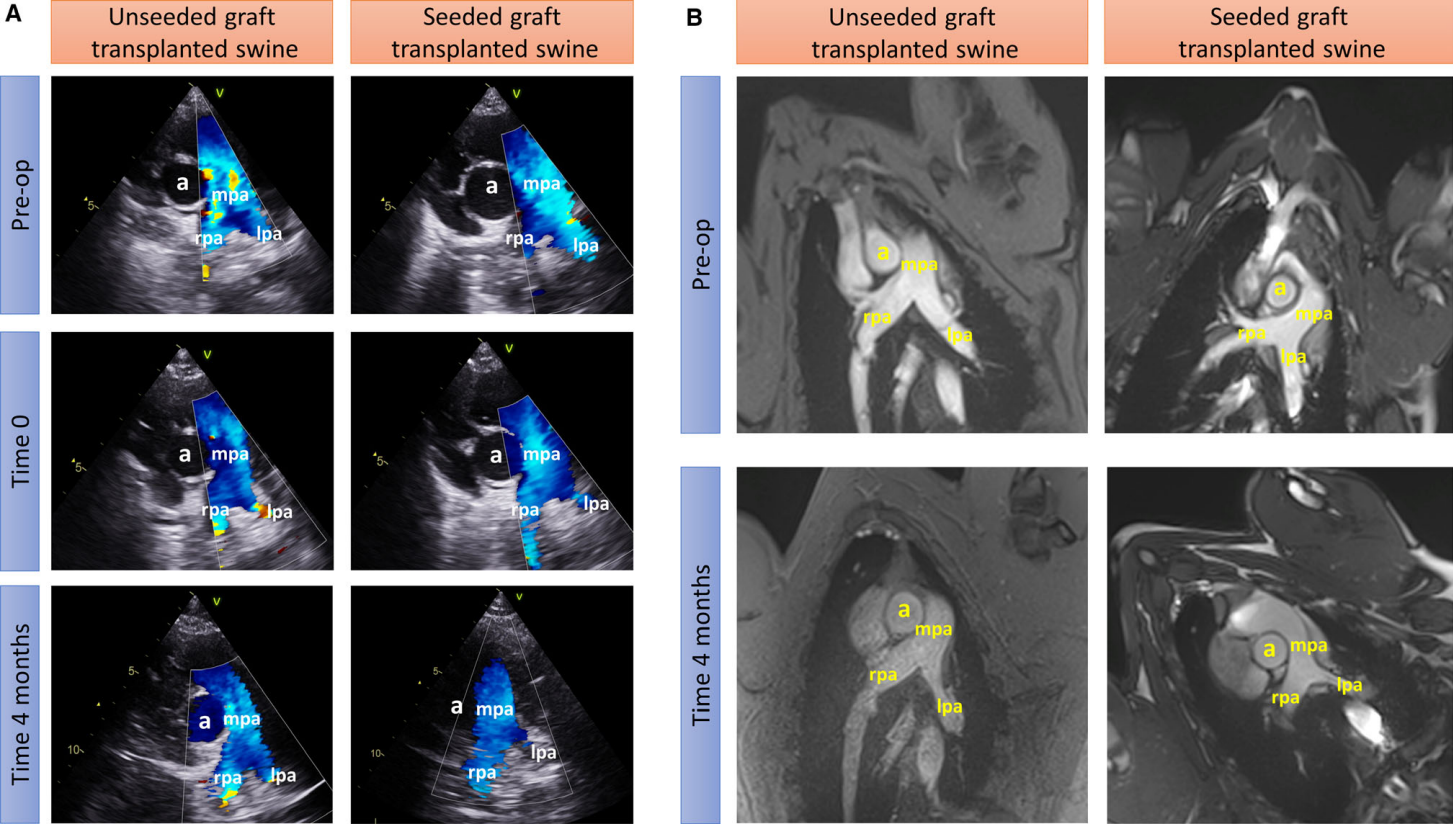
Imaging studies using Doppler echocardiography (**A**) and cardiac MRI (**B**). Vascular structures are highlighted (a) aorta, (mpa) main pulmonary artery, (rpa) right pulmonary artery, and (lpa) left pulmonary artery. Preop indicates preoperation.

Figure 7 shows representative images of Doppler and cardiac magnetic resonance, which confirm the patency of grafted LPA in both piglets transplanted with seeded or unseeded grafts. Table 2 reports the individual blood flow velocity at different times of follow‐up, with values within the normal range for both animals.

**Table 2:** Blood Flow Velocity Measured by Doppler

	Unseeded	Seeded
LPA		
Preop	0.86	1.23
Postop	0.80	1.41
RPA		
Preop	1.33	0.93
Postop	0.89	1.26
MPA		
Preop	1.04	1.33
Postop	1.12	1.35

Values are mL/sec. LPA indicates left pulmonary artery; MPA, main pulmonary artery; Postop, postoperation; Preop, preoperation; RPA, right pulmonary artery.

- Histological analysis of native PA and implanted grafts

**Figure 8:**
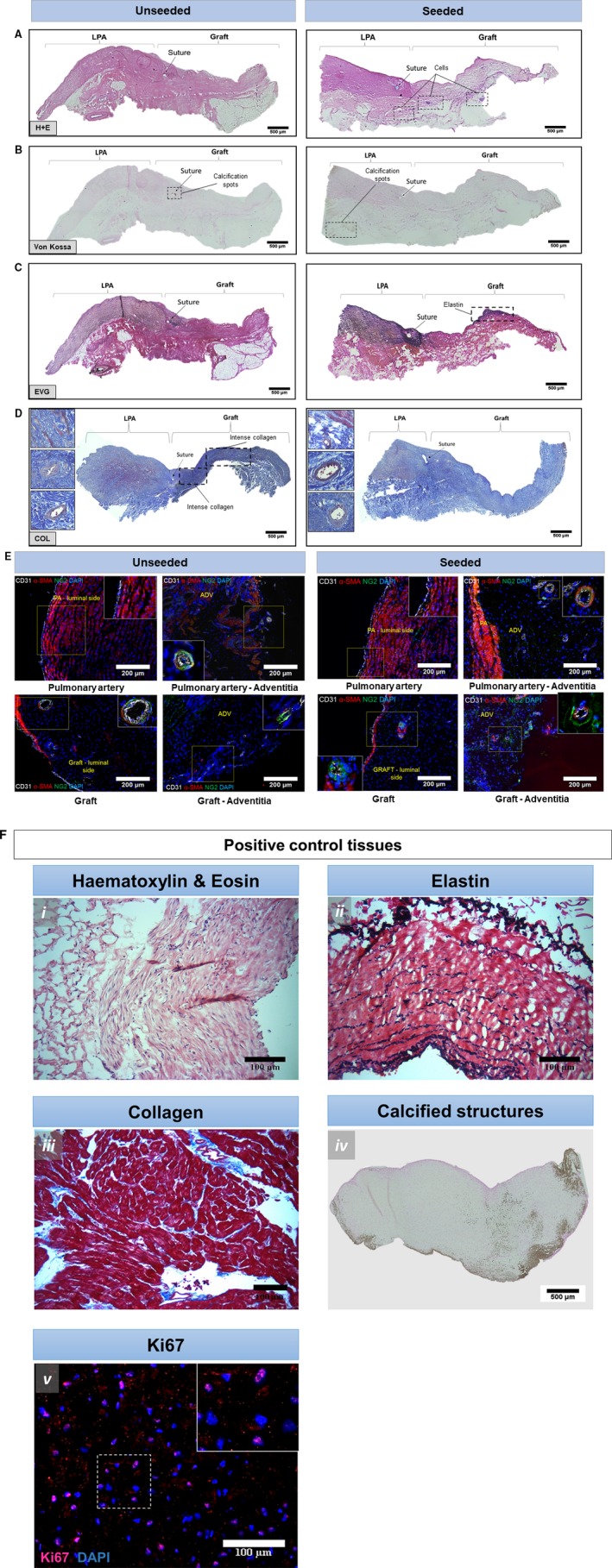
Analyses of the explanted grafts. **A**, H&E staining of unseeded and seeded grafts. **B**, Von Kossa staining. **C**, Von Gieson staining. **D**, Collagen staining. **E**, Immunohistochemistry of LPA and explanted grafts. Cells expressing α‐SMA (red) and CD31 (white) are present in the internal layer and luminal site of the native tissue and graft. In addition, NG2‐positive cells (green) were identified in the adventitia around the vasa vasorum. **F**, Positive control tissues for immunohistochemistry graft comparison. Swine saphenous vein showing H&E (i) and EVG staining (ii, cells and elastic fibers, respectively). Swine MI heart biopsy displaying Azan Mallory staining (collagen) in the remote area (iii). Aortic valve from swine MI model showing Von Kossa staining for identification of microcalcifications (iv). Swine health cardiac tissue stained with Ki67 for cell proliferation (v). ADV and PA indicate adventitia and pulmonary artery (yellow color); CD, cluster of differentiation; COL, collagen; DAPI, 4′,6‐diamidin‐2‐fenilindolo; EVG, elastic fibers Van Giesons’; H+E, hematoxylin and eosin; LPA, left pulmonary artery; MI, myocardial infarction; NG2, neural/glial antigen 2; SMA, smooth muscle actin.

Hematoxylin and eosin images show extensive nucleation throughout the structure of the proximal and distal seeded graft (Figure 8A). In both seeded and unseeded grafts, there were minimal levels of calcification, as assessed using von Kossa staining (Figure 8B). Elastin is a major component of the PA. In line with this, van Gieson staining displayed a consistent layer of organized elastin in the native tissue of the LPA (Figure 8C). Elastin deposits were visible in the seeded graft attached to the proximal and distal LPA, but less defined in the unseeded graft (Figure 8C). Collagen fibers (both interstitial and perivascular) were detected especially in the unseeded explant, probably attributable to the formation of a fibrous scarred tissue. In addition, new muscle tissue appeared in the LPA and in the perivascular area of the explants (Figure 8D). The immunohistochemical analysis showed an organized multilayer of smooth muscle cells populating the tunica media of the LPA. Endothelial cells identified by the CD31 marker were present at the luminal side of the artery and in vessels within the adventitia (Figure 8E). Cells expressing NG2 were also found in the adventitia of the vessels. Notably, more organized vessel structures appeared in seeded graft adventitia compared with unseeded ones (Figure 8E). Positive staining controls, used in association with main histological analyses of grafts, are shown in Figure 8F.

## Discussion

Cell therapy and tissue engineering are gaining momentum for correction of congenital heart defects.^12^, ^13^, ^14^ Preclinical studies have demonstrated the utility of delivering different cell types, including skeletal myoblasts, cord blood stem cells, and mesenchymal stromal cells, either intramyo‐ or epicardially to treat right ventricle dysfunction and pulmonary artery hypertension induced by pressure or volume overload.^15^, ^16^, ^17^ This experimental evidence was followed by clinical trials, some already concluded and others still ongoing, in patients with hypoplastic left heart syndrome reviewed in a previous work.^18^ A more‐complex endeavor aims to create cellularized conduits to correct severe cardiac defects, such as tetralogy of Fallot, which are characterized by a constriction of the right ventricle outflow tract and PA. Initial evidence indicates that acellular grafts repopulated with ECs or bone marrow cells are less susceptible to thrombosis or obstruction.^4^, ^5^, ^6^ We recently developed an alternative approach where a swine small intestinal submucosa graft functionalized with umbilical mesenchymal stromal cell‐derived vascular smooth muscle cells was used for replacement of the PA in piglets.^11^After 6 months from implantation, grafted arteries had developed a functional intima and media without evidence of aneurism formation. Using a similar strategy, we have now demonstrated the feasibility of using CPs to generate living vascular conduits for reconstruction of the PA. The 2 approaches are mutually compatible and adaptable to the individual clinical condition and time of diagnosis. Fetal cell or umbilical cord stem cells isolated at the time of birth could be used if CHD is diagnosed prenatally. When diagnosis of CHD is made after birth or in babies who require a palliative intervention soon after birth, tissue‐specific cardiac cells, such as CPs, could be isolated from surgical cardiac leftovers to generate living conduits to be implanted at the occasion of the definitive correction.^3^

Human pericytes represent the cell product to be ideally tested in preclinical safety/efficacy studies before a first‐in‐human clinical trial. However, their use in a xenogeneic piglet model is discouraged because of the adverse effects and confounding influence of immunosuppression to prevent rejection. These concerns are particularly serious considering the need for chronic immunosuppression to be started in young developing animals. Therefore, the use of a swine equivalent product is a sensible way forward. Regulatory agencies are usually reluctant to allowing an animal‐equivalent product where possible, but, under these circumstances, the Medicines & Healthcare products Regulatory Agency has provided us with a favorable feedback for swine cells to be used in piglets for the model rather than human cells in swine. In addition, the Medicines & Healthcare products Regulatory Agency's opinion is that the PA reconstruction used here is an appropriate in vivo model to assess feasibility and efficacy of the cell‐engineered CorMatrix conduit.

The surrogate cell product was validated through extensive characterization of antigenic markers, using in situ immunohistochemistry, immunocytochemistry, and flow cytometry. We also confirmed the clonogenic capacity of freshly processed or thawed single‐sorted sPCs. The possibility of generating frozen stock of CPs provides therapeutic flexibility for meeting the surgical schedule (ie, surgery happening at a later time than the one required for the preparation of the graft) or multistage surgical corrections. In addition, swine and human pericytes share the ability to recruit ECs and promote endothelial network formation through secretion of paracrine factors.^3^ Pericytes signal to ECs through the binding of ANG1 to Tie2, resulting in activation of pathways mediating survival, proliferation, migration, and anti‐inflammatory signals. Using a Tie2 antagonist, here we demonstrated that this signaling is instrumental to the recruitment of ECs by the sCP secretome. Our data indicate that sCPs promote the organization of sPAECs on Matrigel, likely through activation of migration and inhibition of degradation. This is in line with the well‐known role of pericytes acting as vascular stabilizers of nascent vascularization.

The use of ECM as a biological scaffold for tissue repair and regeneration is an established procedure within the clinical community. Commercial scaffolds manufactured from a range of ECM materials from different animals are available for use in cardiac surgery. CorMatrix, the most used product of this type, is a decellularized porcine small intestinal submucosa product that has received approval by both the US Food and Drug Administration and European authorities for cardiovascular applications. A recent meta‐analysis assessing the results of clinical studies using CorMatrix in pediatric populations indicates favorable outcomes.^19^ However, some of the claimed advantages of the material, such as the bioinductive capacity to support the ingrowth of reparative cells from the host without inducing inflammatory reactions, have been questioned by several reports. Histological examination of failing grafts showed moderate‐to‐severe grade inflammation in the explant tissue without signs of regeneration or integration.^20^, ^21^, ^22^ Interestingly, these reactions were not observed in swine models, thus suggesting the possibility that inflammation was a reaction to antigenic epitopes present in the swine intestine, but not in humans.^22^ Moreover, 90% of the CorMatrix is made of collagen type I, but contains little amounts of elastin,^23^ which is instead a major component of a pulmonary artery and a determinant in maintaining vascular hemodynamic efficiency.^24^

Recellularization with stromal cells before in vivo implantation could overcome the inadequate constructive remodeling capacity and lack of elasticity of the native cross‐linked CorMatrix. Confirming the data of our previous study on human pericytes,^3^ the present report shows that sCPs proliferate and engraft onto the external surface of CorMatrix, maintaining a high degree of viability and the original antigenic phenotype. This is in keeping with the goal to create a cellularized adventitial layer, from which, upon in vivo implantation, sCPs could exert paracrine recruiting effects on the recipient's cells and timely remodeling of the matrix. The lack of sCP penetration into the matrix core before in vivo implantation is, in our opinion, a positive outcome. In fact, for sPCs to infiltrate the internal layer of the conduit, the matrix should undergo degradation. This might result in making the conduit unable to withstand the mechanical forces in vivo. The equilibrium between breakdown of the structure attributed to proteases and production of ECM proteins plays a fundamental role in the stabilization and rearrangement of the graft as well as native blood vessel.^25^ Our results indicate that collagen content progressively increased in sCP‐seeded grafts throughout the maturation in the bioreactor. Collagen possesses useful mechanical properties that can confer the CorMatrix with increased elasticity and greater extensibility before fracture. In line with the above, mechanical tests at different times of the in vitro maturation process demonstrated that CorMatrix conduits acquired a more elastic behavior when seeded with sCPs, as denoted by a significant decrease in Young's modulus, but also an increased strain at rupture. Similar mechanical effects were reported by Sodian et al in a study using tissue‐engineered trileaflet heart valves.^26^

The in vivo study provided us with valuable data in preparation of a properly designed efficacy trial, but also highlighted the strengths and weaknesses of the swine model. We used Doppler and cardiac magnetic resonance imaging to measure blood flow velocity and confirm the patency of the implanted graft. The values of blood flow velocity from the present study are consistent with a previous report.^11^ A power calculation based on combined data from the 2 studies indicate that a minimum of 7 animals per group would be necessary to detect a difference of 0.3 mL/min with an SD of 0.2. Our previous study confirmed that graft stenosis is a rare event in swine compared with humans,^11^ possibly attributable, as mentioned above, to a lower inflammatory reaction to a product from the same species. Therefore, hemodynamic outcomes should be considered in combination with histological outcomes, such as matrix remodeling, endothelialization of the intima, and cellularization of the media and adventitia, as shown in the present report.

## Conclusions

We demonstrated that sCPs can be used as a cell surrogate of human CPs to engineer CorMatrix conduits implantable in the piglet's LPA. A controlled randomized efficacy/safety study based on this technology is currently ongoing with provision to be completed by March 2020. The completion of the full study would provide clear evidence of the advantage of using pericytes‐engineered conduits over the unseeded CorMatrix, in terms of structural and functional performance. This novel approach, using cells extracted from small pieces of cardiac tissue available during palliative surgery, may open new avenues for correction of congenital cardiac defects with remarkable medical and social benefits.

## Sources of Funding

This study was supported by grants from the Sir Jules Thorn Charitable Trust (Caputo, Madeddu), the Enid Linder Foundation (Caputo, Ghorbel), the British Heart Foundation (Caputo), the National Institute for Health Research (NIHR) Bristol Biomedical Research Centre in Cardiovascular Medicine (Caputo), and the Medical Research Council (Madeddu, Caputo, Ghorbel).

## Disclosures

None.

## Supporting information


**Data S1.** XXXX.
**Table S1.** Code of Donor Neonatal Swine and Analyses Performed on Corresponding Cardiac Samples and Isolated Cells
**Table S2.** Antibodies Used in Immunohistochemistry and Immunocytochemistry Studies
**Table S3.** Antibodies Used in Flow Cytometry Studies
**Table S4.** TaqMan Probes Used in the Molecular Biology Studies
**Table S5.** Analyses Performed on sCP‐Engineered Grafts
