# Supplementary material for: In Vitro and In Vivo Preclinical Testing of Pericyte‐Engineered Grafts for the Correction of Congenital Heart Defects
Source: J Am Heart Assoc. 2020 Feb 11;9(4):e014214. doi: 10.1161/JAHA.119.014214 (PMC7070228; doi:10.1161/JAHA.119.014214)
Supplement: Supplementary file 1 — Data S1. XXXX. Table S1. Code of Donor Neonatal Swine and Analyses Performed on Corresponding Cardiac Samples and Isolated Cells Table S2. Antibodies Used in Immunohistochemistry and Immunocytochemistry Studies Table S3. Antibodies Used in Flow Cytometry Studies Table S4. TaqMan Probes Used in the Molecular Biology Studies Table S5. Analyses Performed on sCP‐Engineered Grafts [file JAH3-9-e014214-s001.pdf]

Supplemental Material

Data S1.

Harvesting and processing of cardiac tissue

Four-weeks-old Large white/Landrace piglets (UK registered breeder) were euthanized with Euthatal, and heart samples were harvested for *in situ* identification and isolation/expansion of swine cardiac pericytes (sCPs) (**Table S1**). Peripheral blood was collected *via* an indwelling jugular vein cannula for isolation of peripheral blood mononuclear cells (PBMNCs) and extraction of serum to be used for sCP expansion. In some experiments, commercial swine heat-inactivated serum (Sigma-Aldrich, UK) was used in alternative to the piglet serum.

***In situ* immunohistochemistry of sCPs**

Cardiac tissue samples were collected and fixed in 4% (w/v) PFA (Sigma-Aldrich, UK) for 16 hours, at +4°C. After one wash in 1x Phosphate Buffered saline (PBS), fixed samples were placed in 30% (w/v) Sucrose for 48 hours, at +4°C, and embedded in OCT (O.C.T. Compound, VWR, UK, cat n: 361603E). Eight-micron sections were cut and fixed in ice-cold glacial Acetone (Fisher, UK) for 5 min, at -20°C. After fixation, sections were kept air-dried for 20 min and hydrated in 1x PBS. 0.01% (v/v). Triton X-100 (Sigma-Aldrich, UK) was used for the membrane permeabilization for 10 min, at RT. Non-specific binding sites were blocked using 5% (v/v), foetal bovine serum (FBS, Life Technologies, UK) in 1x PBS as blocking solution. Double staining combinations of anti-swine NG2/anti-human CD31 and anti-swine CD34/anti-human CD31 primary antibodies were used. After a 16-hour incubation at +4°C, sections were washed in 1xPBS, and secondary antibodies were incubated on the sections for 1 hour, at RT, in the dark (**Table S2**). The nuclei were counterstained with DAPI (Sigma-Aldrich). The slides were mounted using Fluoromount-G® mounting media (Sigma-Aldrich) and immunofluorescent images were taken using x10, x20 objectives of Zeiss Observer.Z1 microsope (Carl Zeiss Microscopy, LLC, US) and Zen pro software.

CP isolation and expansion

Cardiac samples were processed using a modification of the GMP-compliant standard operating procedure (SOP) previously employed for isolation/expansion and characterization of CPs from the neonatal human heart.[3] Briefly, single cell suspensions of immunomagnetically-sorted $CD31^-$ $CD34^+$ cells were cultured onto dishes coated with 1% (w/v) swine gelatin (Sigma-Aldrich) containing Endothelial Cell Growth medium-2 (EGM-2, Promocell, UK) supplemented with 10% (v/v) heat-inactivated swine serum (Sigma-Aldrich), and 1% (v/v) Penicillin and Streptomycin (Pen/Strep). Once reached 80-90% confluence, primary colonies were passaged to new culture dishes. At P2, cells were split for further expansion or generation of frozen stocks.

Assessment of sCP characteristics

- ***Immunocytochemistry***

Expanded cells (N=7 biological replicates run in triplicate) were fixed with freshly prepared 4% (w/v) PFA in 1x PBS for 10 min at +4°C , washed with 1x PBS and probed with the indicated antibodies (**Table S2**). For detection of intracellular antigens, cells were permeabilized for 10 min at +4°C with 0.1% (v/v) Triton X-100 (Sigma-Aldrich) diluted in 1x PBS. Cells were incubated with the indicated primary antibodies (16 hours at +4°C) and appropriate secondary antibodies (1:200 anti-rabbit Alexa 488 or 1:200 anti-mouse Alexa 488, 1 hour at +20°C in the dark). The nuclei were counterstained with DAPI (Sigma-Aldrich). Slides were mounted using Fluoromount-G (Sigma-Aldrich). Cells were analyzed at a 200X and 400X magnification. Zeiss Observer.Z1 and Zen pro software were utilized to compose and overlay the images. Swine Pulmonary Artery Endothelial Cells (sPAECs, AMSBio, USA) were used as positive controls for endothelial markers.

- ***Flow cytometry***

Cells ($1x10^6$, N=3 biological replicates) were washed in 1X DPBS (Life Technologies) and treated with 1x trypsin/EDTA (Life Technologies). Then, they were washed, spun at 400xg for 10 min at +4°C and re-suspended in FACS-staining buffer containing 0.1% (w/v) BSA, 1mM EDTA and 0.1% (v/v) sodium azide, followed by blocking of unspecific binding with 10% (w/v) BSA in 1X DPBS. Next, $2x10^5$ cells/tube were incubated with antibodies for 30 min at +4°C, in the dark (**Table S3**). For detection of the endothelial marker CD31, cells were washed twice in FACS buffer. The flow cytometry procedure included Fluorescence Minus One (FMO) controls. sPAECs were used to verify the expression of endothelial antigens. In addition, we used the Fixable Viability Dye eFluor 780 (eBioscience, UK) to label dead cells and exclude them from the gating strategy. Prior to sample acquisition, the cells were fixed with 1% (v/v) PFA in 1x PBS.

- ***Clonogenic assay:***

The test was performed on two sCP lines at P3, using a motorized device connected to the flow cytometric sorter (Cyclone, Beckman Coulter, US). Sorted cells were placed into each well of a 96-well culture plate (Greiner Bio-one, UK) and cultured up to 4 weeks in EGM-2 for quantification of colonies generated from a single cell. A comparative assay between fresh and frozen-thawed sCPs was performed to assess if both conditions allow the generation of clones.

- ***Quantitative PCR of angiogenic factors***

Total RNA was obtained from cultured sCPs (miRNeasy mini kit, Cat n: 217004, Qiagen) and reverse-transcribed using a High Capacity RNA-to-cDNA Kit (Cat n: 4387406, Life Technologies). The reverse transcription–PCR was performed using the first-strand cDNA with TaqMan Fast Universal PCR Master Mix (Cat n: 4324018, Life Technologies) and on a Quant Studio 6 Flex Real-Time PCR system (Applied Biosystems) for the genes specified in **Table S4**.

- ***Secretion of angiogenic factors***

Dedicated anti-human ELISA kits were used to measure the immunoreactive levels of Vascular Endothelial Growth Factor-A (VEGF-A), Angiopoietin 1 (ANG 1), Angiopoietin 2 (ANG 2), and basic Fibroblast Growth Factor (bFGF) proteins in conditioned media (CM) from sCPs, which were cultured for 48 hours in 2.5 mL serum-free, endothelial basal medium 2 (EBM-2) under normoxia. All the ELISA kits were from R&D System (cat n: DY293B, DY923, DY623, DY233-05, respectively). SPAECs were used as a control. The optical density (OD) of each well was determined using a Dynex Opsys MR microplate reader (Aspect Scientific, UK) set to 450 nm.

- ***Endothelial network formation***

The capacity of cells to form networks on Matrigel was assessed using sCPs or sPAECs alone or both in coculture. In addition, the network formation capacity of sPAECs was assessed following stimulation with sCP-CM or unconditioned media (UCM). First, sCPs were labelled with the long-term cell tracker Dil (Thermo fisher cat n: C7001). Cells were seeded on top of 70 µL thick-coated Matrigel (BD Biosciences) in EGM2 for 6 hours. Images were taken under bright field at 5× and the length of the networks was measured.

- ***Chemotactic activity***

Here, we tested the capacity of the sCP-CM to induce the migration of sPAECs. The latter were seeded on transwell cell culture inserts equipped with 8 µm pore size polycarbonate membranes (Corning, cat n: 3422) in EBM-2 basal medium. The migration of sPAECs was stimulated by adding 500 µL of sCP-CM to the bottom of the same wells. In parallel, EBM-2 basal medium or EBM-2 supplemented with 100 ng/mL of swine recombinant VEGF-A (Cambridge Bioscience, cat n: RP0403S-005) were used to assess spontaneous and growth factor directed migration. In separate assays, an antagonist of Tie-2 kinase receptor (Abcam, cat n: 141270) was used to contrast the effect of the sCP-CM on migration. After 16-hour incubation at +37°C, the polycarbonate membranes were washed and scraped with a cotton swab to remove non-migrating cells. Migrated cells at the bottom side of the filter were assessed by GIEMSA and DAPI. For GIEMSA staining,

the membranes were fixed in 4% (w/v) PFA and 100% (v/v) Methanol. GIEMSA stain solution was added for 60 min at RT, protected from light. For staining of nuclei, the membranes were washed in 4% (w/v) PFA and incubated with 1:1000 DAPI for 2 min at RT. In either case, membranes were washed twice and gently removed from the inserts with a scalpel for the visualization of the cells and nuclei, respectively. Membranes were analyzed with an epifluorescence microscope at ×200 magnification; 10 fields were randomly acquired, and cells counted. Migrated cells were expressed as a percentage of total seeded cells.

- ***Endothelial cell proliferation***

The capacity of sPAECs to proliferate in the presence of sCPs and sCP-CM was assessed by Click-iT™ EdU (5-ethynyl-2'-deoxyuridine) Cell Proliferation Kit for Imaging, Alexa Fluor™ 488 dye (Thermo fisher, cat n: C10337) using UCM and sCP-CM media. Briefly, sCPs were labelled with Dil according the manufacturer's instructions prior to seeding for the assay. Swine PAECs and sCPs were seeded into plastic dishes and the EdU was incubated for a minimum of 16 hours with EBM-2 and sCP-CM. Cells were fixed in 4% (w/v) PFA and the membrane were permeabilized using 0.1% (v/v) Triton X-100. The EdU detection reagents were added to the wells for 30 minutes and an anti-swine CD31 primary antibody (Abcam cat n: ab28364) was incubated overnight, at +4 °C, protected from light. A secondary antibody was used to detect sPAECs stained with CD31 antigen. Cells were incubated with 1:10,000 HOECHST for 3 minutes, at +21 °C, protected from light. Fluorescent images were taken with a fluorescent microscope and 10 fields were acquired randomly using 20X magnification objective. Proliferative sPAECs were counted as a percentage of total sPAECs seeded.

Studies on cellularized CorMatrix

- ***Static culture of sCPs on CorMatrix***

Pieces of CorMatrix® ECM® (CorMatrix Cardiovascular, Sunnyvale, CA) were cut, firmly positioned at the bottom of wells of a 48-well plate using Cell Crown inserts (Sigma-Aldrich), and primed with EGM-2 media for 48 hours. Next, sCPs (P5, 20,000/cm2) were seeded into each CorMatrix-containing well and cultured for 5 days. The CM was then collected and the CorMatrix samples were cut in three pieces: one was fixed in 4% (w/v) PFA for 16 hours at +4°C and embedded in paraffin for histological studies, while the other two pieces were OCT-frozen for molecular biology analyses. Eight biological replicates were examined unless differently specified.

- ***Dynamic culture of sCPs on CorMatrix***

After completion of the 5-day static culture, sCP-seeded CorMatrix was further grown in a 3D CulturePro™ Bioreactor (TA instruments, USA). Non-seeded CorMatrix was used as control. The conduit was stitched to the rotating arm of the Bioreactor and stitched back onto itself to fashion a tube shape through the centre of which runs the rotating arm. The bioreactor was filled with EGM-2 media supplemented with 10% (v/v) swine serum and 1% (v/v) Pen/Strep and maintained in the incubator at +37°C. Seven and fourteen days later, the CM were collected, and the conduits were unstitched and cut in three pieces: one was fixed in 4% (w/v) PFA for 16 hours at +4°C and embedded in paraffin, and the two other pieces were frozen for OCT-embedding and for RNA and protein analysis. Four biological replicates were examined unless otherwise specified. The list of swine and specific experimental usage of derived sCP lines in the CorMatrix studies is reported in **Table S5.**

- ***Characterization of cellularized CorMatrix grafts***

<u>*Histological assessment of the graft structure:*</u> To obtain a general layout of the cells, elastin, and interstitial collagen distribution within the CorMatrix grafts, frozen sections were stained with Haematoxylin and Eosin (H&E), Elastic tissue-Van Gieson's (EVG), and Mallory's trichrome using a Shandon Varistain 24-4 slide stainer (Thermofisher, UK) on slides mounted with DPX (Sigma-Aldrich) and covered with cover slips. Images were acquired using Zeiss Axio

Observer.Z1 with Zen Blue software. Collagen deposits onto grafts were quantified as percentage of total area using ImageJ software.
Viability and apoptosis: Cells were detached from the CorMatrix graft by enzymatic digestion and cytospun for 5 min at 500 rpm at RT onto histology slides using an EZ double cytofunnelTM (Thermofisher, UK cat n: A78710005) and a cytospin machine (Cytospin 4, Thermofisher, UK). Two different enzymes (Trypsin and Accutase) were used for cell detachment. Viability was determined with Trypan blue and apoptosis with Apoptag Red In situ Apoptosis Detection Kit (Millipore, UK cat n: S7165). As a positive control for apoptosis, sCPs were treated with 15 µm H2O2 (Sigma, UK cat n: H1009) for 1 hour at +37°C. In addition, cell viability was assessed *in situ* (without detaching cells from the graft) at 5 days post-static and 7 days post-dynamic culture using a Viability/Cytotoxicity immunofluorescent kit (Thermofisher, UK, cat n: L3224). Saponin (Sigma-Aldrich) treated samples were used as positive controls. Grafts were imaged using Zeiss microscope.
Proliferation: After permeabilization with 0.1% (v/v) Triton X-100, samples were stained with anti-swine Ki67 primary antibody (Abcam, UK, cat n#: ab15580).
Expression of mural cell markers: Here, we determined the effect of engraftment on the expression of pericyte and Vascular Smooth Muscle Cell (VSMC) markers. Eight-micron sections from frozen samples were cut and fixed in ice-cold glacial Acetone (Thermofisher) for 5 min at -20°C. After fixation, sections were kept air-dried for 20 min and hydrated in 1x PBS. Non-specific binding sites were blocked using 5% (v/v) foetal bovine serum (FBS Life Technologies, UK) or 10% (v/v) Normal Goat serum (NGS, Life Technologies) in 1x PBS as blocking solution. Sections were incubated for 16 hours, at +4°C, with an anti-swine NG2 primary antibody or with anti-human and anti-swine α-Smooth Muscle Actin (α-SMA, Sigma, UK), Calponin (CALP, Abcam, UK), Transgelin (TAGLN, Santa-Cruz, UK), Smoothelin (SMTN, Santa Cruz, UK), and Smooth Muscle Myosin Heavy Chain (SMMHC, Abcam, UK). Secondary antibodies were incubated on the sections for 1 hour, at RT, protected from the light. The nuclei were counterstained with DAPI. The slides were mounted using Fluoromount-G® mounting media (Sigma-Aldrich) and immunofluorescent and brightfield images were taken using x2.5, x10, x20 objectives of Zeiss microscope.
Analysis of collagen secretion by CorMatrix grafts: The CM of cellularized grafts (collected after culture under static or dynamic conditions) were centrifuged at 400 *g* for 10 min at RT to assess the levels of soluble Collagen 1 (COLIA1) using an anti-human COLIA1 ELISA kit (R&D Systems, cat n: DY6220-05). The OD value was measured using a microplate reader set to 450 nm.
Mechanical tests of CorMatrix grafts: The elastic modulus, maximum tensile stress and strain at rupture were measured using an Instron 3343 device (Illinois Tool Works Inc., USA). The study was conducted on unseeded and cellularized grafts (N=3 biological replicates) and a swine Left Pulmonary Artery (LPA) specimen serving as control. In detail, CorMatrix tubular structures, seeded and unseeded, were opened along the stitches and cut to obtain a rectangular-shaped sample with length of 20±5mm, width of 6.5±0.35mm and thickness of 3.5±0.15mm. Tests were performed at +37ºC in PBS, the distance between the two clamps was set to 10±1mm and specimens were subjected to strain rate of 0.01 min^{-1} until failure. Stress–strain curves of grafts and swine LPA (control) were derived from the load-elongation data.

In vivo study assessing the feasibility of implanting a cellularized graft conduit in piglet LPA
Surgical procedures were performed with swine under general anesthesia (Ketamine/Midazolam/Dexmedetomidine, Isoflurane) and neuromuscular blockade (Pancuronium Bromide). Details of the operation have been reported previously.[11] Briefly, a left posterolateral thoracotomy was performed in two 4-week-old sister Landrace female piglets. The proximal and distal part of the LPA (just before the upper and middle lobe branches of the LPA)

was clamped and a 3–4 mm of the LPA was resected to accommodate the conduit-shaped graft (~10 mm long and ~6 mm diameter). One piglet received a conduit cellularized with sCPs from a sister piglet and cultured under static (5 days) and dynamic condition (7 days). The other piglet was implanted with an unseeded conduit. Animals recovered under intense postoperative monitoring for the initial 24 hours. Analgesic (Paracetamol, Morphine) and antibiotics (Cefuroxime) were administered during this period according to the needs.

Imaging studies were performed using a two-dimensional Doppler Echocardiography system (VividQ, GE Healthcare, UK) and a cardiac magnetic resonance 3-T scanner (Siemens Healthcare, Erlangen, Germany) at baseline and 4 months after implantation. Then, swine were euthanized by an overdose of IV pentobarbitone according to the surgical facility standard protocols. The grafted LPA was harvested and stored in PBS on ice until transported to the laboratory. Tissues were either snap frozen in OCT or fixed in 4% (w/v) PFA before OCT or paraffin inclusion and histology analysis.

Immunohistochemistry of vascular graft conduits

For a general overview of the grafted LPA structure, we performed H&E and elastin staining. Azan Mallory staining was used for the detection of fibrosis in the explants. The occurrence of microcalcifications was assessed using Von Kossa (Millipore, UK, cat n: 1.00362.0001). Endothelial cells in the intima and mural cells in the tunica media were recognized using antibodies reported in **Table S2**. Images were captured using a Zeiss Axio Observer.Z1 equipped with a Zen Blue software.

Statistical analyses

Average values are plotted with group size value shown in figure legends. Statistical significance for differences between experimental groups was determined using Student's t-test when comparing two groups and ANOVA with post-hoc when comparing more than two groups. Values were expressed as means±standard error of the mean (SEM) or standard deviation (SD). Probability values (P) <0.05 were considered significant. Results from the *in vivo* feasibility study are reported in a descriptive format.

Table S1. Code of donor neonatal swine and analyses performed on corresponding cardiac samples and isolated cells.

Swine code	Weight (g)	Experimental use
240617A	0.9	Immunohisto-cytochemistry, PCR, ELISA, Migration assay w/o anti-Tie2
080717A	0.08	Immunohisto-cytochemistry, PCR, Matrigel-co, Migration assay w/o anti-Tie2
080717B	0.08	Immunocytochemistry, FACS, PCR, Matrigel-co/CM, ELISA, Migration assay without anti Tie2, EdU assay
060917A	0.09	Immunocytochemistry, Growth curve- Viability-DT, FACS, Clonogenic assay, PCR, Matrigel-co, ELISA
060917B	0.09	Growth curve- Viability-DT, FACS, Matrigel-co/CM, ELISA, Migration assay without anti-Tie2, EdU assay
060917C	0.09	Immunocytochemistry, Growth curve -Viability-DT, Clonogenic assay
140618A	0.2	PCR, Matrigel-CM, Migration assay-anti-Tie2, EdU assay
190618A	0.013	Immunocytochemistry, Matrigel-CM, Migration assay-anti-Tie2, EdU assay
240718A	0.01	Immunocytochemistry

Table S2. Antibodies used in immunohistochemistry and immunocytochemistry studies.

Marker	Technique	Permeabilization	Reactivity	Primary antibody source and dilution	Secondary antibody source
α-SMA	ICC-IF, IHC-IF	Yes	Human/Swine	Sigma, 1:400	Cy3-conjugated
Calponin	ICC-IF, IHC-IF	Yes	Human/swine	Abcam, 1:100	Invitrogen, A488 Goat α-Rabbit
CD31	ICC-IF	Yes	Swine	Abcam, 1:20	Invitrogen, A488 Goat α-Rabbit
CD31	IHC-IF	No	Human	R&D, 1:50	Invitrogen A647 highly adsorbed Goat α-mouse
CD34	IHC-IF	Yes	Swine	Abcam, 1:100	Invitrogen A488 Goat α-Rabbit
CD73	ICC-IF	Yes	Swine	R&D, 1:10	Invitrogen, APC donkey anti-sheep
CD146	ICC-IF	Yes	Human/Swine	Abcam, 1:100	Invitrogen, A488 Goat α-Rabbit
GATA-4	ICC-IF	Yes	Human	Abcam, 1:100	Invitrogen, A488 Goat α-rabbit
NANOG	ICC-IF	Yes	Human	Abcam, 1:100	Invitrogen, A488 Goat α-mouse
NG2	ICC-IF, IHC-IF	Yes	Swine	NovusBiological, 1:50	Invitrogen, A488 Goat α-Rabbit
OCT-4	ICC-IF	Yes	Human/Swine	Abcam, 1:100	Invitrogen, A488 Goat α-Rabbit
PDGFRβ	ICC-IF	Yes	Swine	GeneTex, 1:100	Invitrogen, A488 Goat α-Mouse
SMMHC11	ICC-IF, IHC-IF	Yes	Human/swine	Abcam, 1:100	Invitrogen, A488 Goat α-Rabbit
Smoothelin	ICC-IF, IHC-IF	Yes	Human/swine	Santa-Cruz, 1:100	Invitrogen, A488 Goat α-Mouse
SOX2	ICC-IF	Yes	Human/Swine	Merck Millipore, 1:100	Invitrogen, A488 Goat α-Rabbit
Transgelin	ICC-IF, IHC-IF	Yes	Human	Santa-Cruz, 1:50	Invitrogen, A488 Goat α-Mouse
VE-cadherin	ICC-IF	Yes	Human/Swine	Santa Cruz, 1:50	Invitrogen, A488 Goat α-Mouse
Vimentin	ICC-IF	Yes	Human	Abcam, 1:400	Invitrogen, A488 Goat α-Rabbit

.

Table S3. Antibodies used in flow cytometry studies.

Marker	Permeabilization	Reactivity	Primary antibody source dilution	Fluorophores
CD31	No	Swine	Serotec, 1:80	PE
CD44	No	Swine	eBioscience, 1:20	APC
CD45	No	Swine	Serotec, 1:25	FITC
CD73	No	Swine	R&D, 1:10	Invitrogen, APC donkey anti-sheep
CD90	No	Swine	eBioscience, 1:20	Pe-Cy7
CD105	No	Swine	LifeSpan, 1:5	PE
CD146	No	Swine	Serotec, 1:60	FITC
PDGFRβ	No	Human	Biolegend, 1:25	PE
Dye eFluor 780	No		eBioscience, 1:1000	APC-Cy7

Table S4. TaqMan probes used in the molecular biology studies.

Gene	Species	Assay ID
ANG1	Swine	Ss03391075_m1
ANG2	Swine	Ss03392362_m1
PPIA (housekeeper)	Swine	Ss03394782_g1
VEGF-A	Swine	Ss0339390_m1

Table S5. Analyses performed on sCP-engineered grafts.

Swine code	Post-static	Post-dynamic	In vivo
240617A	H&E, EVG, Collagen, Proliferation, Apoptosis, NG2 and VSMC markers	H&E, EVG, Collagen, Proliferation, Apoptosis, NG2, VSMC markers, and Mechanical tests	
080717A	Collagen		
080717B	Viability, H&E, EVG, Collagen, Proliferation, Apoptosis, NG2 and VSMC markers	Viability, H&E, EVG, Collagen, Proliferation, Apoptosis, NG2 and VSMC markers	
060917A	H&E, EVG, Collagen, Proliferation, Apoptosis, NG2 and VSMC markers	H&E, EVG, Collagen, Proliferation, Apoptosis, NG2, VSMC markers, and Mechanical tests	
060917B	H&E, EVG, Collagen, Proliferation, Apoptosis, NG2 and VSMC markers	H&E, EVG, Collagen, Proliferation, Apoptosis, NG2, VSMC markers, and Mechanical tests	
191217A	Viability		
140618A	Viability, H&E, EVG, Collagen, Proliferation, Apoptosis, NG2 and VSMC markers	Viability	Engraftment
190618A	Viability, H&E, EVG, Collagen, Proliferation, Apoptosis, NG2 and VSMC markers	Viability	Engraftment
240718A	Viability, H&E, EVG, Collagen, Proliferation, Apoptosis, NG2 and VSMC markers	Viability	Engraftment
050918A	H&E, EVG, Collagen, Proliferation, Apoptosis, NG2 and VSMC markers		Engraftment
050918B	H&E, EVG, Collagen, Proliferation, Apoptosis, NG2 and VSMC markers		Engraftment
30.01.19A			Engraftment
30.01.19 B	Viability	Viability	Engraftment
Unseeded Graft	Viability, H&E, EVG, Collagen, Proliferation, Apoptosis, NG2 and VSMC markers	Viability, H&E, EVG, Collagen, Proliferation, Apoptosis, NG2, VSMC markers, and Mechanical tests	Engraftment
